# Levetiracetam Suppresses the Infiltration of Neutrophils and Monocytes and Downregulates Many Inflammatory Cytokines during Epileptogenesis in Pilocarpine-Induced Status Epilepticus Mice

**DOI:** 10.3390/ijms23147671

**Published:** 2022-07-12

**Authors:** Taira Matsuo, Rie Komori, Minami Nakatani, Shiori Ochi, Aya Yokota-Nakatsuma, Junichi Matsumoto, Fuyuko Takata, Shinya Dohgu, Yasuhiro Ishihara, Kouichi Itoh

**Affiliations:** 1 Laboratory for Pharmacotherapy and Experimental Neurology, Kagawa School of Pharmaceutical Sciences, Tokushima Bunri University, 1314-1 Shido, Sanuki 769-2193, Japan; t-matsuo@kph.bunri-u.ac.jp (T.M.); komorir@kph.bunri-u.ac.jp (R.K.); s158055@stu.bunri-u.ac.jp (M.N.); shiori.ochi.1117@gmail.com (S.O.); 2 Laboratory of Immunology, Kagawa School of Pharmaceutical Sciences, Tokushima Bunri University, 1314-1 Shido, Sanuki 769-2193, Japan; yokotate@kph.bunri-u.ac.jp; 3 Department of Pharmaceutical Care and Health Sciences, Faculty of Pharmaceutical Sciences, Fukuoka University, Fukuoka 814-0180, Japan; jmatsumoto@fukuoka-u.ac.jp (J.M.); ftakata@fukuoka-u.ac.jp (F.T.); dohgu@fukuoka-u.ac.jp (S.D.); 4 Program of Biomedical Science, Graduate School of Integrated Sciences for Life, Hiroshima University, Hiroshima 739-8521, Japan; ishiyasu@hiroshima-u.ac.jp

**Keywords:** levetiracetam, epileptogenesis, neuroinflammation, neutrophil

## Abstract

Acute brain inflammation after status epilepticus (SE) is involved in blood–brain barrier (BBB) dysfunction and brain edema, which cause the development of post-SE symptomatic epilepsy. Using pilocarpine-induced SE mice, we previously reported that treatment with levetiracetam (LEV) after SE suppresses increased expression levels of proinflammatory mediators during epileptogenesis and prevents the development of spontaneous recurrent seizures. However, it remains unclear how LEV suppresses neuroinflammation after SE. In this study, we demonstrated that LEV suppressed the infiltration of CD11b^+^CD45^high^ cells into the brain after SE. CD11b^+^CD45^high^ cells appeared in the hippocampus between 1 and 4 days after SE and contained Ly6G^+^Ly6C^+^ and Ly6G^−^Ly6C^+^ cells. Ly6G^+^Ly6C^+^ cells expressed higher levels of proinflammatory cytokines such as IL-1β and TNFα suggesting that these cells were inflammatory neutrophils. Depletion of peripheral Ly6G^+^Ly6C^+^ cells prior to SE by anti-Ly6G antibody (NIMP-R14) treatment completely suppressed the infiltration of Ly6G^+^Ly6C^+^ cells into the brain. Proteome analysis revealed the downregulation of a variety of inflammatory cytokines, which exhibited increased expression in the post-SE hippocampus. These results suggest that Ly6G^+^Ly6C^+^ neutrophils are involved in the induction of acute brain inflammation after SE. The proteome expression profile of the hippocampus treated with LEV after SE was similar to that after NIMP-R14 treatment. Therefore, LEV may prevent acute brain inflammation after SE by suppressing inflammatory neutrophil infiltration.

## 1. Introduction

Status epilepticus (SE) is a serious medical emergency that leads to death or permanent neurologic injury and can result in the development of symptomatic epilepsy [1]. Accordingly, the management of SE is important to prevent mortality and the development of post-SE symptomatic epilepsy. Various clinical trials have indicated that conventional antiepileptic drugs (AEDs) (e.g., phenobarbital, valproate, diazepam or phenytoin) suppress acute seizures, but none of these drugs prevent the development of post-SE epilepsy [2,3,4]. Therefore, to suppress epileptogenesis, a new perspective on therapeutic drugs is needed.

Recent studies suggest that inflammation in the brain is a crucial mechanism underlying the pathology of epilepsy [5,6]. Brain-resident immune cells, microglia, are major contributors to neuroinflammation and the proliferation of activated resident microglia contributes to neuronal death in the hippocampus after SE [7,8,9,10]. In addition to microglia, infiltrating immune cells from peripheral tissues contribute significantly to pathogenesis after SE [7,11]. It has also been reported that suppression of post-SE neuroinflammation by blocking prostaglandin receptor EP2 and inhibiting IL-1β biosynthesis can provide neuroprotection, prevent blood–brain barrier (BBB) dysfunction, alleviate morbidity and rescue behavioral deficits [12,13,14]. Hence, molecules that suppress neuroinflammation are prospective candidates for suppressing epileptogenesis.

Levetiracetam (LEV) is an established second-generation AED that exerts broad-spectrum antiepileptic effects and is widely used to treat focal onset and generalized seizures [15]. We have previously reported that LEV suppresses the occurrence of spontaneous recurrent seizures after pilocarpine-induced SE [16]. LEV also suppressed increased expression levels of proinflammatory mediators and prevented BBB dysfunction during a latency period after SE [17]. The target brain binding site of LEV is synaptic vesicle glycoprotein 2A (SV2A), which is a prototype synaptic vesicle protein regulating action potential-dependent neurotransmitter release [18,19]. SV2A is a possible target for LEV to suppress seizure generation, but we have shown that LEV can suppress the activation of BV-2 microglia that do not express SV2A, suggesting the existence of other target(s) of LEV [20]. Furthermore, the mechanism by which LEV suppresses neuroinflammation also remains unclear.

In this study, using a pilocarpine-induced SE model, we demonstrated that the infiltration of leukocytes into the brain, most of which were Ly6G^+^Ly6C^+^ neutrophils and Ly6G^−^Ly6C^+^ monocytes, was significantly suppressed by LEV treatment. In addition, we compared the expression profiles of various cytokines in the hippocampus after SE in LEV-treated and neutrophil-depleted mice. Many cytokines with increased post-SE expression showed a decrease in both. Our findings suggest that the mechanism by which LEV suppresses neuroinflammation might be due to the suppression of neutrophil infiltration.

## 2. Results

### 2.1. CD11b^+^CD45^high^ Cells Infiltrate the Brain after Pilocarpine-Induced Status Epilepticus (SE)

**Figure 1:**
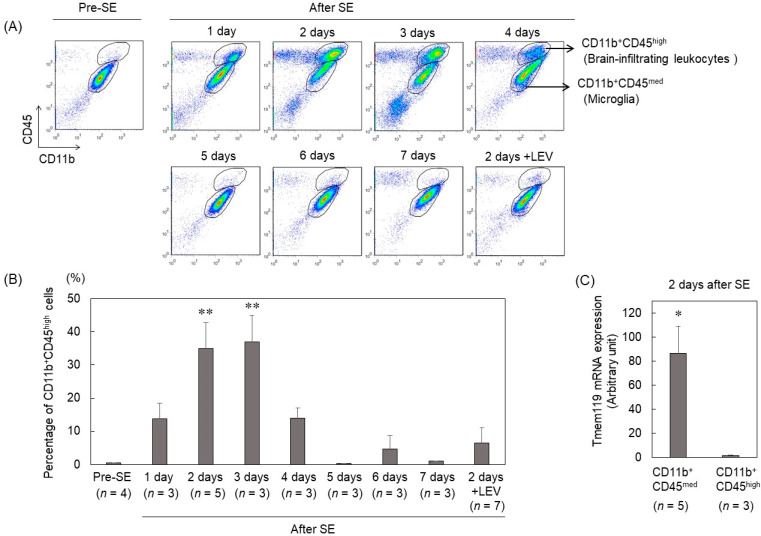
The extent and dynamics of brain-infiltrating leukocytes after SE. (**A**) Flow cytometry analysis of the immune cell fractions isolated from the hippocampus. Mice were killed 1–7 days after pilocarpine-induced SE, and immune cell fractions were prepared as described in the Materials and Methods section. Debris (SSC-A/FSC-A) and doublets (FSC-H/FSC-A and SSC-H/SSC-A) were excluded, and dead cells were identified using propidium iodide (PI). Brain-infiltrating leukocytes were distinguished from microglia based on the expression of CD45. The results are representative of at least 3 independent experiments. (**B**) Changes as a function of time in the percentage of CD11b^+^CD45^high^ cells within the PI^−^ population defined by flow cytometry analysis. *n* indicates the number of independent experiments. Data are means ± SEMs. The data were analyzed using ANOVA followed by Dunnett’s test. ** *p* < 0.01 vs. Pre-SE. (**C**) Mice were killed 2 days after pilocarpine-induced SE. mRNA was isolated from FACS-sorted CD11b^+^CD45^med^ and CD11b^+^CD45^high^ cells and measured by qRT–PCR. The expression of Tmem119 was normalized to that of Actb. *n* indicates the number of independent experiments. Data are means ± SEMs. * *p* < 0.05, unpaired *t* test.

To investigate the temporal change in brain-infiltrating leukocytes after pilocarpine-induced SE, immune cell fractions were isolated from the hippocampus using density gradient centrifugation techniques from 1 to 7 d after SE and stained with anti-CD45 and anti-CD11b antibodies for flow cytometry. In pre-SE mice, only the CD11b^+^CD45^med^ cell subset was observed in the immune cell fraction from the hippocampus. The CD45^high^ cell subset (CD11b^+^CD45^high^) was observed in addition to the CD11b^+^CD45^med^ cell subset in mice that entered SE (Figure 1A). The number of CD11b^+^CD45^high^ cells peaked 2–3 d after SE and almost disappeared 5 d after SE (Figure 1B). We isolated each subset from the hippocampus 2 d after SE using FACS and examined the expression of Tmem119, which is a specific microglial marker. CD11b^+^CD45^med^ cells expressed higher levels of Tmem119 (Figure 1C). CD45 is expressed on all nucleated hematopoietic cells, and CD11b is expressed on many leukocytes including monocytes and neutrophils. Previous studies have reported that the expression profiles of CD11b and CD45 markers can distinguish microglia (CD11b^+^CD45^med^) from infiltrating leukocytes (CD11b^+^CD45^high^) [21,22,23]. Therefore, our results indicated that CD11b^+^CD45^med^ cells and CD11b^+^CD45^high^ cells are microglia and brain-infiltrating leukocytes, respectively.

### 2.2. Characterization of CD11b^+^CD45^high^ Infiltrating Cells

**Figure 2:**
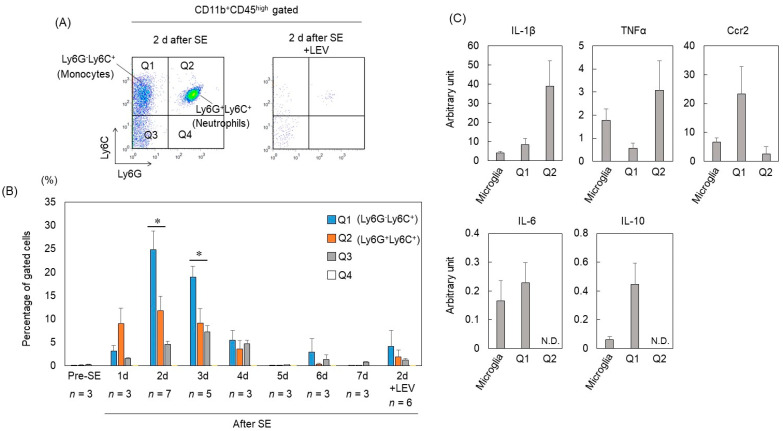
Characterization of CD11b^+^CD45^high^ brain-infiltrating leukocytes. (**A**) Flow cytometry analysis of CD11b^+^CD45^high^ fractions 2 d after SE. As shown in Figure 1, debris, doublets and dead cells were excluded. Live cells were subgated on CD11b^+^CD45^high^ cells. Ly6C/Ly6G immunoreactivity was used to distinguish neutrophils and monocytes. The results are representative of at least 3 independent experiments. (**B**) Changes as a function of time in the percentage of cells in the Q1, Q2, Q3 and Q4 fractions within the PI^−^ population defined by flow cytometry analysis. *n* indicates the number of independent experiments. Data are means ± SEMs. * *p* < 0.05, unpaired *t* test. (**C**) Mice were killed 2 days after pilocarpine-induced SE. mRNA was isolated from FACS-sorted CD11b^+^CD45^med^ (microglia), CD11b^+^CD45^high^ Q1 (monocytes) and CD11b^+^CD45^high^ Q2 (neutrophils) cells and measured by qRT–PCR. The expression of each gene was normalized to the expression of Actb. Number of samples: microglia, *n* = 5; Q1, *n* = 4; Q2, *n* = 4. Data are means ± SEMs. N.D., not detected.

To explore in detail the characterization of CD11b^+^CD45^high^ infiltrating cells, we investigated the expression of Ly6G and Ly6C in the CD11b^+^CD45^high^ fraction by flow cytometry. Ly6G and Ly6C express granulocytic and monocytic subsets, respectively. Flow cytometry analysis demonstrated that the CD11b^+^CD45^high^ fraction included three subsets (Q1; Ly6G^−^Ly6C^+^, Q2; Ly6G^+^Ly6C^+^, Q3; Ly6G^−^Ly6C^-^) (Figure 2A). Ly6G^+^Ly6C^+^ cells, which are considered neutrophils, accounted for most of the infiltrating cells 1 d after SE, and 2 to 3 d after SE, the number of Ly6G^−^Ly6C^+^ cells, which are considered monocytes, was the highest (Figure 2B). To investigate the expression profile of inflammation-related genes in infiltrating cells, quantitative real-time PCR (qRT–PCR) was performed on FACS-sorted CD11b^+^CD45^med^ (microglia), Ly6G^−^Ly6C^+^ (Q1) and Ly6G^+^Ly6C^+^ (Q2) fractions 2 d after SE. The mRNA levels of proinflammatory cytokines IL-1β and TNFα in the Ly6G^+^Ly6C^+^ fraction were 4-fold and 6-fold higher than those in the Ly6G^−^Ly6C^+^ fraction, respectively. In contrast, the mRNA levels of the anti-inflammatory cytokines IL-6 and IL-10 in the Ly6G^−^Ly6C^+^ fraction were higher than those in the Ly6G^+^Ly6C^+^ fraction (Figure 2C). The Ly6G^−^Ly6C^+^ fraction also showed high expression of CCR2, which is an important chemokine receptor for monocytes to infiltrate the brain after SE [11]. These findings suggested that Ly6G^+^Ly6C^+^ neutrophils that infiltrate the brain early after SE and express proinflammatory cytokines might contribute to acute neuroinflammation, while Ly6G^−^Ly6C^+^ monocytes expressing anti-inflammatory cytokines may contribute to the end of neuroinflammation. 

### 2.3. LEV Suppresses the Infiltration of CD11b^+^CD45^high^ Cells into the Brain and the Expression of Inflammatory Cytokines after SE

We have previously reported that LEV suppressed the increased expression of proinflammatory cytokines such as IL-1β and TNFα in the hippocampus 2 d after SE [17]. However, it remains unclear how LEV suppresses the expression of these genes. It was reported that infiltrating leukocytes contribute to neuroinflammation and pathogenesis after status epilepticus [10,11,24]. Therefore, we investigated the effect of LEV on infiltrating cells in the brain after SE. Interestingly, LEV-treated mice showed little infiltration of CD11b^+^CD45^high^ cells 2 d after SE (Figure 1A,B and Figure 2A,B). 

**Figure 3:**
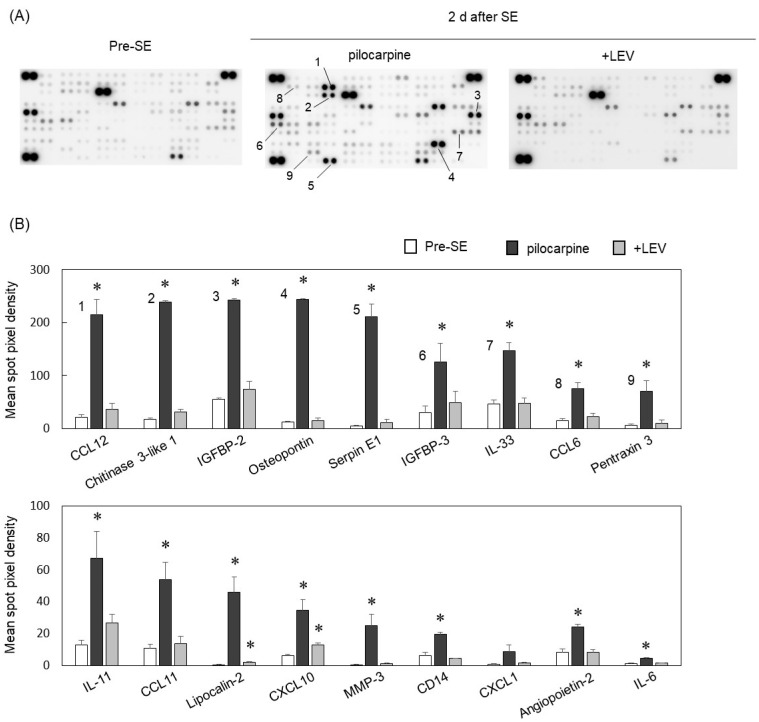
Proteome analysis using cytokine array. (**A**) Mice were killed 2 days after pilocarpine-induced SE. LEV was repeatedly administered for 2 days after the termination of SE by midazolam. Whole hippocampi were lysed with RIPA buffer supplemented with protease inhibitor cocktail. Representative results are shown. The numbers shown in pilocarpine blots correspond to the numbers in bar graph (**B**). (**B**) Pixel intensities of spots were measured using image analysis software (Multi Gauge). Bar graphs show the representative altered proteins. The numbers shown in bar graph correspond to the numbers in Figure 3A. Number of animals: Pre-SE, *n* = 3; pilocarpine, *n* = 4; +LEV, *n* = 3. Data are means ± SEMs. * *p* < 0.05 versus pre-SE mice, unpaired *t* test.

To further investigate the effects of LEV on the hippocampus after SE, we used a Proteome Profiler Mouse XL Cytokine Array that can simultaneously detect 111 soluble mouse inflammatory proteins. Hippocampal tissue lysates from pre-SE mice, mice 2 d after SE and LEV-treated mice after SE were examined (Figure 3A). At 2 d after SE, we found that the expression of 65 cytokines, including 10 interleukins and 12 chemokines, was increased more than 2-fold compared to that in pre-SE mice. Representative proteins with significantly altered expression are shown in Figure 3B. The schematic representation of the Mouse XL Cytokine Array and a cytokine list are shown in Figure S1. Proteome analysis revealed that pilocarpine-induced seizures significantly upregulated the expression of many inflammatory proteins. In the LEV-treated mice 2 d after SE, of the 65 cytokines whose expression was increased more than 2-fold, 44 cytokines were reduced in expression by 50% or less compared with the mice 2 d after SE without LEV administration. Furthermore, the expression levels of many cytokines, including CCL12 and osteopontin (the expression of which was significantly increased), were reduced to the same level as those in pre-SE mice by treatment with LEV. These results suggested that LEV might suppress the increased expression of inflammatory cytokines by inhibiting the infiltration of leukocytes into the brain after SE.

### 2.4. Depletion of Circulating Neutrophils Suppresses Increased Cytokine Expression after SE

**Figure 4:**
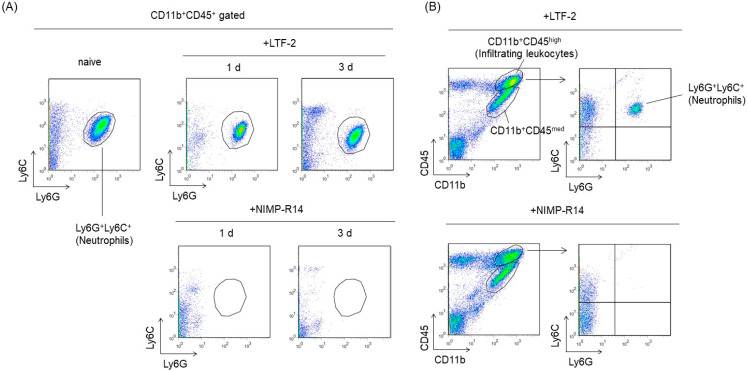

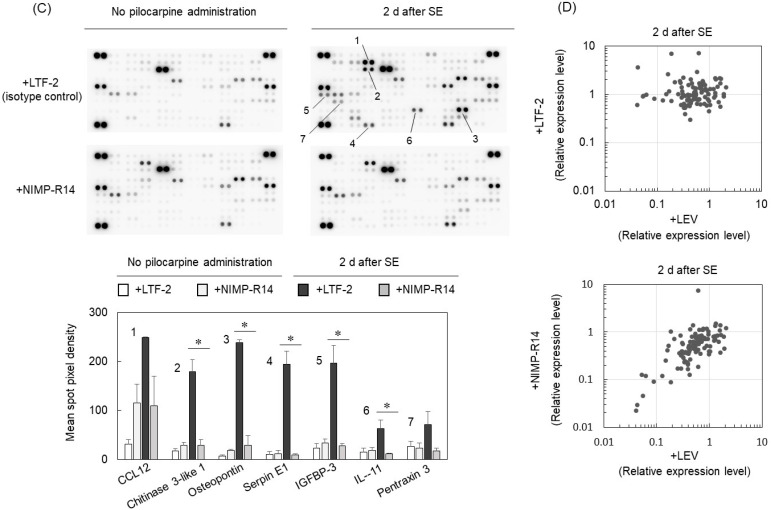
The effects of NIMP-R14 administration on pilocarpine-induced SE. (**A**) Mice were administered an intraperitoneal injection of NIMP-R14 or LTF-2 which is the rat IgG2b isotype control. The Ly6G^+^Ly6C^+^ cell subset in the peripheral blood was evaluated 1 d and 3 d after antibody injection by flow cytometry. The results are representative of 3 independent experiments. (**B**) Mice were administered an intraperitoneal injection of NIMP-R14 or LTF-2 one day prior to pilocarpine administration. At 2 d after SE, mice were killed and immune cell fractions were prepared from the hippocampi for flow cytometry analysis. Brain-infiltrating leukocytes were identified the based on expression of CD11b and CD45. Neutrophils were then identified based on the expression of Ly6G and Ly6C. The results are representative of 4 independent experiments. (**C**) Mice were administered an intraperitoneal injection of NIMP-R14 or LTF-2 one day prior to pilocarpine administration. At 2 d after SE, the lysates of whole hippocampi were analyzed by a Proteome Profiler Mouse XL Cytokine Array kit. The nonpilocarpine-administration groups were killed 3 days after antibody injection, and hippocampal lysates were prepared. The numbers shown in the right panel of LTF-2 correspond to the numbers in the bar graph. Pixel intensities of spots were measured using image analysis software. Bar graphs show the representative altered proteins. *n* = 3 mice per group. Data are means ± SEMs. * *p* < 0.05, unpaired *t* test. (**D**) Cytokine expression patterns were compared between LEV-treated and LTF-2-treated mice (top panel) and between LEV-treated and NIMP-R14-treated mice (bottom panel) with cytokine arrays. The relative expression in mice 2 d after SE was normalized to the mean of LEV-treated or antibody-treated mice from mice treated with pilocarpine only.

To investigate the involvement of brain-infiltrating neutrophils in neuroinflammation, mice were treated with NIMP-R14 antibody prior to administration of pilocarpine. The monoclonal antibody NIMP-R14 is highly specific for murine Ly6G and Ly6C and is useful for the depletion of neutrophils in mice. We confirmed that administration of 200 μg of NIMP-R14 completely depleted Ly6G^+^Ly6C^+^ neutrophils from circulating blood for at least 3 days (Figure 4A). Next, we investigated whether NIMP-R14 suppresses the infiltration of neutrophils into the brain after SE. Flow cytometric analysis demonstrated that there were no Ly6G^+^Ly6C^+^ neutrophils in the hippocampi of mice treated with NIMP-R14 at 2 d after SE (Figure 4B). However, the infiltration of Ly6G^−^Ly6C^+^ monocytes was observed in the hippocampus. To evaluate the effects of Ly6G^+^Ly6C^+^ neutrophil depletion on neuroinflammation after SE, we assessed the expression of cytokines, chemokines and growth factors in hippocampal homogenates using a Proteome Profiler Mouse XL Cytokine Array. In mice treated with NIMP-R14 prior to administration of pilocarpine, the expression levels of many cytokines, such as CCL12, chitinase 3-like 1 and osteopontin, were significantly decreased compared with those in the LTF-2-treated SE-mice (Figure 4C). Among the 65 cytokines that exhibited increased expression 2 d after SE, 40 cytokines were reduced in expression by 50% or less compared with the LTF-2-treated SE-mice. These results suggest that infiltrating Ly6G^+^Ly6C^+^ neutrophils contribute to neuroinflammation after pilocarpine-induced SE. Furthermore, most of the cytokines that were downregulated by NIMP-R14 were consistent with the cytokines that were downregulated by LEV (Figure 4D), suggesting that the anti-inflammatory effect of LEV in the brain may be due to the suppression of neutrophil infiltration.

## 3. Discussion

In this study, we showed that a large number of CD11b^+^CD45^high^ leukocytes, which contained Ly6G^+^Ly6C^+^ neutrophils and Ly6G^−^Ly6C^+^ monocytes, infiltrated the hippocampus 2–3 days after pilocarpine-induced SE. It has been shown that infiltrating immune cells (i.e., neutrophils, monocytes, lymphocytes) are involved in neuroinflammation and contribute to pathogenesis. In a spinal cord injury (SCI) model, two types of cell groups (Ly6G^+^Ly6G^−^ and Ly6G^−^Ly6C^+^) have been reported to infiltrate after injury and contribute to the resolution of acute inflammation and the subsequent tissue repair process [25]. The expression profiles of inflammation-related genes such as IL-1β and TNFα in Ly6G^+^Ly6G^−^ and Ly6G^−^Ly6C^+^ cells are consistent with those of Ly6G^+^Ly6C^+^ and Ly6G^−^Ly6C^+^ cells that we showed in this study. Previous studies also reported that Ly6C^+^CCR2^+^ monocytes contribute to neuroinflammation and morbidity after kainic acid (KA)-induced SE [7,10,11]. Since Ly6G^−^Ly6C^+^ cells express CCR2, Ly6C^+^CCR2^+^ cells, and Ly6G^−^Ly6G^+^ cells are considered to be the same cell subset, these findings support that infiltrating Ly6G^+^Ly6C^+^ and Ly6G^−^Ly6C^+^ cells are involved in neuroinflammation and epileptogenesis after pilocarpine-induced SE.

We have previously reported that consecutive treatment with LEV inhibited the temporarily increased BBB leakage in the hippocampus 2 d after pilocarpine-induced SE [17]. In this study, we demonstrated that LEV strongly inhibited the infiltration of both Ly6G^+^Ly6C^+^ neutrophils and Ly6G^−^Ly6C^+^ monocytes after SE (Figure 1A,B). The infiltrating neutrophils have been shown to produce MMP-9 and IL-1β, which are involved in BBB dysfunction [26,27,28] and contribute to pathogenesis in animal models of neurological disease, such as the experimental autoimmune encephalomyelitis (EAE) model of multiple sclerosis [29,30], intracerebral hemorrhage [31] and ischemic stroke [32]. In the pilocarpine-induced SE model, we showed the infiltration of Ly6G^+^Ly6C^+^ neutrophils and the increased expression of multiple cytokines associated with BBB dysfunction, such as MMP-3 and MMP-9, in the early phases of epileptogenesis after SE (Figure S2). Furthermore, antibody-mediated depletion of peripheral neutrophils suppressed the expression of various cytokines, including MMP-3 and MMP-9. In addition, we investigated whether LEV protected BBB functions using an in vitro BBB model and showed that LEV suppressed the permeability of Evans blue albumin through brain capillary endothelial cells (Figure S3). These findings suggest that infiltrating neutrophils may play a main role in BBB dysfunction in the acute phase of epileptogenesis and that LEV treatment may contribute to suppressing neutrophil infiltration by BBB dysfunction.

Astrocytes play an important role in maintaining central nervous system function by releasing gliotransmitters, including glutamate, D-serine and ATP, and many reports show that astrocytes contribute to the pathophysiology of epilepsy [33,34,35]. JNK signaling is involved in reactive astrogliosis and decreased resilience to kainic acid-induced seizures [35,36]. We comprehensively investigated gene expression in the hippocampi of pilocarpine-induced SE mice and revealed increased expression of GFAP and decreased expression of glutamate transporter genes (SLC1A2 and SLC1A3) 2 d after SE, which suggests the presence of reactive astrocytes (Komori, R., under revised in IJMS). A recent study showed that TNFα induces enhanced phosphorylation of JNK and an increase in HDAC2, as well as a decrease in SLC1A2 in cultured astrocytes [37]. TNFα produced by infiltrating Ly6G^+^Ly6C^+^ neutrophils may contribute to astrogliosis after SE. Further experiments are needed to elucidate the interaction between astrocytes and infiltrating cells in the brain.

Infiltrating Ly6C^high^ monocytes have been reported to promote neuroinflammation and exacerbate neuronal damage after SE [7,11]. We showed in this study that neuroinflammation was suppressed by inhibiting the infiltration of neutrophils, but neuronal cell death has not yet been evaluated. Since LEV suppresses the infiltration of both neutrophils and monocytes, the inhibition of monocyte infiltration by LEV may contribute to preventing neuronal death after SE. Further experiments are needed to elucidate the anti-epileptogenic mechanism of LEV.

Using a pilocarpine-induced SE model, we have reported that LEV clearly suppresses the incidence of convulsive seizures after SE, but carbamazepine and valproate exhibit nearly no effects on seizures after SE [20]. It has also been reported that prophylactic treatment with carbamazepine, phenytoin and valproate has little efficacy on preventing the recurrent chronic seizures after traumatic brain injury [38]. Several basic studies of prophylactic treatment with AEDs in post-SE epilepsy/seizures (PSEE/S) have been reported [39]. Moreover, several clinical studies for the prophylactic treatment with AEDs in PSEE/S, poststroke SE, and posttraumatic epilepsy SE have been reported [40,41,42]. Our investigations of LEV in the pilocarpine-SE model suggest that prophylactic treatment with LEV after SE, including brain damage, may decrease the risk of developing epilepsy (spontaneous recurrent seizures) [16,17]. 

A prospective observational study showed that LEV is more effective in controlling postoperative seizures than other AEDs [43]. Kundu et al. reported the efficacy of surgical approaches for the treatment of temporal lobe epilepsy [44]. The historical literature has shown that approximately 60% of patients who undergo fornicotomy, with or without anterior commissurotomy, have some seizure control benefit. Further studies on the antiepileptic and anti-epileptogenic mechanisms of LEV are needed, but the combination of LEV and surgery may be effective in the treatment of refractory epilepsy.

In conclusion, the inhibition of neutrophil and monocyte infiltration into the hippocampus by LEV treatment may be involved in the suppression of brain inflammation and the incidence of spontaneous recurrent seizures induced by SE. Clarifying the effects of LEV treatment on the balance between the infiltration of neutrophils and monocytes into the brain and BBB failure during the acute phase post-SE will open new avenues for understanding the mechanism of acquired epilepsy and may be key to developing new therapies.

## 4. Materials and Methods

### 4.1. Experimental Animals

The protocols for all animal experiments were approved by the Tokushima Bunri University Animal Care Committees and were performed in accordance with the National Institutes of Health (Bethesda, MD, USA) Animal Care and Use Protocol. All efforts were made to minimize the number of animals used and their suffering. Six-week-old male ICR mice were purchased from Japan SLC (Shizuoka, Japan). All mice were maintained with laboratory chow and water ad libitum on a 12 h light/dark cycle.

### 4.2. Pilocarpine-Induced Status Epilepticus (SE) Model and Seizure Assessment

The pilocarpine-induced SE model was established according to our previous report [16,17,20]. ICR mice (8–10 weeks old) were injected with methyl scopolamine (1 mg/kg, Sigma–Aldrich, St Louis, MO, USA) intraperitoneally (i.p.). Then, a single dose of pilocarpine (Sigma–Aldrich) was administered (290 mg/kg, i.p.). Animals were placed in a plastic chamber (10 × 15 × 30 cm), and their behavior was observed before and after pilocarpine injection. SE was defined as the incidence of five generalized convulsive seizures for a maximum time of 60 min [45]. To terminate SE, we injected all mice with midazolam (MDZ, 1 mg/kg, i.p.; Alfresa Pharma, Osaka, Japan) once or more as needed.

### 4.3. Administration of LEV and Antibodies

The dose of LEV (LKT Labs, St. Paul, MN, USA) used in this study was 360 mg/kg. LEV dissolved in distilled water was orally administered at an injection volume of 0.1 mL/10 g of body weight within 30 min after MDZ injection and thereafter twice a day (at 8:30 and 17:30). For in vivo neutrophil depletion, mice were injected intraperitoneally with 200 μg of the Ly6G antibody NIMP-R14 (Bio X cell, Lebanon, NH, USA) 24 h before the injection of pilocarpine. LTF-2 (Bio X cell, Lebanon, NH, USA) (200 μg) was used as a rat IgG2b isotype control. LTF-2 is a monoclonal antibody that reacts with keyhole limpet hemocyanin.

### 4.4. Isolation of Immune Cell Fractions from the Hippocampus and Analysis of Microglia and Infiltrating Leukocytes

Mice were perfused with PBS under isoflurane anesthesia and the hippocampi were isolated and collected in ice-cold HBSS. In the case of the sample for RNA extraction, 2 to 3 hippocampi were combined into one sample to obtain a sufficient number of cells. Subsequently, the hippocampi were dissected, minced and digested for 45 min at 37 °C in the presence of 400 U/mL collagenase (Sigma–Aldrich, St Louis, MO, USA) and 30 U/mL DNase I (Thermo Fisher Scientific, Waltham, MA, USA) in DMEM supplemented with 5% FCS. The cell suspension was pipetted with a fire-polished pasture pipet and passed through a 40 μm cell strainer to achieve a single-cell suspension. The cells were pelleted by centrifugation at 500× *g* for 10 min at 4 °C. The pellet was resuspended in a 37% isotonic Percoll (GE Healthcare, Chicago, IL, USA) and carefully layered on top of 70% isotonic Percoll, followed by centrifugation at 400× *g* for 30 min. The immune cells containing microglia and leukocytes were collected from the boundary between the 37% and 70% Percoll and blocked with anti-mouse CD16/CD32 against Fc receptors (BD Biosciences, San Jose, CA, USA) for 10 min on ice. Cells were then incubated for 60 min in Cell Staining Buffer (Biolegend, San Diego, CA, USA) with anti-CD11b-FITC (1/100, Biolegend, San Diego, CA, USA), anti-CD45-PE (1/100, Biolegend, San Diego, CA, USA), anti-Ly6G-APC (1/100, Biolegend, San Diego, CA, USA) and anti-Ly6C-APC/Cy7 (1/100, Biolegend, San Diego, CA, USA) on ice. Dead cells were stained with propidium iodide. Stained cells were analyzed with a FACSCanto (BD Biosciences, San Jose, CA, USA) or sorted with a FACSAriaII (BD Biosciences, San Jose, CA, USA). Data were analyzed with FlowJo (FlowJo LLC, Ashland, OR, USA).

### 4.5. Flow Cytometry Analysis of Blood Samples

Mice were anesthetized with pentobarbital (50 mg/kg i.p.). Blood was collected by cardiac puncture. After red blood cells were removed with RBC Lysis Buffer (Biolegend, San Diego, CA, USA), the resulting suspension was pelleted by centrifugation and washed twice in PBS. Cells were blocked with anti-mouse CD16/CD32 antibody and stained with anti-CD11b-FITC (1/100), anti-CD45-PE (1/100), anti-Ly6G-APC (1/100) and anti-Ly6C-APC/Cy7 (1/100). Dead cells were stained with propidium iodide. Stained cells were analyzed with a FACSAriaII.

### 4.6. RNA Isolation and Real-Time PCR

Total RNA was isolated from FACS-sorted CD11b^+^CD45^med^, CD11b^+^CD45^high^, Ly6G^+^Ly6C^+^ and Ly6G^−^Ly6C^+^ cells using NucleoSpin RNA XS (Macherey-Nagel, Düren, Germany) or RNeasy Micro Kit (QIAGEN, Venlo, The Netherlands) according to the manufacturer’s instructions. RNA was reverse transcribed to cDNA using ReverTra Ace (ToYoBo, Osaka, Japan). Quantitative real-time PCR was performed using QuantStudio 7 (Applied Biosystems, Norwalk, CT, USA) with Power SYBR Green Master Mix (Applied Biosystems, Norwalk, CT, USA). The primer sequences used in this study are listed in Table 1. The mRNA expression of each gene was normalized to the level of β-actin mRNA for each sample as a standard and expressed as an arbitrary unit.

### 4.7. Proteome Profiler Mouse Cytokine Array

Whole hippocampi were lysed with RIPA buffer (FUJIFILM Wako Pure Chemical Co., Osaka, Japan) supplemented with complete protease inhibitor cocktail (Roche, Basel, Switzerland). Protein concentration was measured with a protein bicinchoninic acid (BCA) kit (TaKaRa, Kusatsu, Japan). Samples were analyzed with a Mouse XL Cytokine Array Kit (R&D Systems, Minneapolis, MN, USA), according to the manufacturer’s instructions. Immunospots were captured with LAS-3000 (Fujifilm, Tokyo, Japan), and data were analyzed with Multi Gauge software (Fujifilm, Tokyo, Japan).

### 4.8. Statistical Analysis

All of the data are presented as the mean ± standard error (S.E.). Student’s *t* test was used to determine the significance of differences between two groups. For comparison of differences among three groups or more, Dunnett’s test, after ANOVA, was adopted. *p* values < 0.05 were considered to indicate statistical significance.

## Figures and Tables

**Table 1:** Primers for real-time PCR.

Target	Forward Primer	Reverse Primer
Actb	CTAGGCACCAGGGTGTGATG	GGGGTACTTCAGGGTCAGGA
Tmem119	GTGTCTAACAGGCCCCAGAA	AGCCACGTGGTATCAAGGAG
IL-1β	GGCTGCTTCCAAACCTTTGA	ACGGGAAAGACACAGGTAGC
TNFα	ACGTGGAACTGGCAGAAGAG	GACCGATCACCCCGAAGTTC
Ccr2	TCCCTGGTATTCATCTTTGG	TTATGTTCCCAAAGACCCAC
IL-6	CTTCCATCCAGTTGCCTTCT	AATTAAGCCTCCGACTTGTG
IL-10	CTCTTACTGACTGGCATGAG	GTATGTTGTCCAGCTGGTCC

## Data Availability

Not applicable.

## Supplementary Materials

The following supporting information can be downloaded at: https://www.mdpi.com/article/10.3390/ijms23147671/s1.

## References

[1] Jacobs M.P., Leblanc G.G., Brooks-Kayal A., Jensen F.E., Lowenstein D.H., Noebels J.L., Spencer D.D., Swann J.W. (2009). Curing epilepsy: Progress and future directions. Epilepsy Behav..

[2] Temkin N.R. (2003). Risk factors for posttraumatic seizures in adults. Epilepsia.

[3] Temkin N.R. (2009). Preventing and treating posttraumatic seizures: The human experience. Epilepsia.

[4] Temkin N.R. (2001). Antiepileptogenesis and seizure prevention trials with antiepileptic drugs: Meta-analysis of controlled trials. Epilepsia.

[5] Vezzani A. (2014). Epilepsy and inflammation in the brain: Overview and pathophysiology. Epilepsy Curr..

[6] Eyo U.B., Murugan M., Wu L.J. (2017). Microglia-Neuron Communication in Epilepsy. Glia.

[7] Tian D.-S., Peng J., Murugan M., Feng L.-J., Liu J.-L., Eyo U., Zhou L.-J., Mogilevsky R., Wang W., Wu L.-J. (2017). Chemokine CCL2–CCR2 Signaling Induces Neuronal Cell Death via STAT3 Activation and IL-1β Production after Status Epilepticus. J. Neurosci..

[8] Avignone E., Ulmann L., Levavasseur F., Rassendren F., Audinat E. (2008). Status epilepticus induces a particular microglial activation state characterized by enhanced purinergic signaling. J. Neurosci..

[9] Bosco D.B., Zheng J., Xu Z., Peng J., Eyo U.B., Tang K., Yan C., Huang J., Feng L., Wu G. (2018). RNAseq analysis of hippocampal microglia after kainic acid-induced seizures. Mol. Brain.

[10] Feng L., Murugan M., Bosco D.B., Liu Y., Peng J., Worrell G.A., Wang H., Ta L.E., Richardson J., Shen Y. (2019). Microglial proliferation and monocyte infiltration contribute to microgliosis following status epilepticus. Glia.

[11] Varvel N.H., Neher J.J., Bosch A., Wang W., Ransohoff R.M., Miller R.J., Dingledine R. (2016). Infiltrating monocytes promote brain inflammation and exacerbate neuronal damage after status epilepticus. Proc. Natl. Acad. Sci. USA.

[12] Jiang J., Quan Y., Ganesh T., Pouliot W.A., Dudek F.E., Dingledine R. (2013). Inhibition of the prostaglandin receptor EP2 following status epilepticus reduces delayed mortality and brain inflammation. Proc. Natl. Acad. Sci. USA.

[13] Maroso M., Balosso S., Ravizza T., Iori V., Wright C.I., French J., Vezzani A. (2011). Interleukin-1beta biosynthesis inhibition reduces acute seizures and drug resistant chronic epileptic activity in mice. Neurotherapeutics.

[14] Rojas A., Ganesh T., Lelutiu N., Gueorguieva P., Dingledine R. (2015). Inhibition of the prostaglandin EP2 receptor is neuroprotective and accelerates functional recovery in a rat model of organophosphorus induced status epilepticus. Neuropharmacology.

[15] Lyseng-Williamson K.A. (2011). Levetiracetam: A review of its use in epilepsy. Drugs.

[16] Itoh K., Inamine M., Oshima W., Kotani M., Chiba Y., Ueno M., Ishihara Y. (2015). Prevention of status epilepticus-induced brain edema and neuronal cell loss by repeated treatment with high-dose levetiracetam. Brain Res..

[17] Itoh K., Ishihara Y., Komori R., Nochi H., Taniguchi R., Chiba Y., Ueno M., Takata-Tsuji F., Dohgu S., Kataoka Y. (2016). Levetiracetam treatment influences blood-brain barrier failure associated with angiogenesis and inflammatory responses in the acute phase of epileptogenesis in post-status epilepticus mice. Brain Res..

[18] Lynch B.A., Lambeng N., Nocka K., Kensel-Hammes P., Bajjalieh S.M., Matagne A., Fuks B. (2004). The synaptic vesicle protein SV2A is the binding site for the antiepileptic drug levetiracetam. Proc. Natl. Acad. Sci. USA.

[19] Meehan A.L., Yang X., Yuan L.L., Rothman S.M. (2012). Levetiracetam has an activity-dependent effect on inhibitory transmission. Epilepsia.

[20] Itoh K., Taniguchi R., Matsuo T., Oguro A., Vogel C.F.A., Yamazaki T., Ishihara Y. (2019). Suppressive effects of levetiracetam on neuroinflammation and phagocytic microglia: A comparative study of levetiracetam, valproate and carbamazepine. Neurosci. Lett..

[21] Martin E., El-Behi M., Fontaine B., Delarasse C. (2017). Analysis of Microglia and Monocyte-derived Macrophages from the Central Nervous System by Flow Cytometry. J. Vis. Exp..

[22] Brandenburg S., Blank A., Bungert A.D., Vajkoczy P. (2020). Distinction of Microglia and Macrophages in Glioblastoma: Close Relatives, Different Tasks?. Int. J. Mol. Sci..

[23] Martin E., Boucher C., Fontaine B., Delarasse C. (2016). Distinct inflammatory phenotypes of microglia and monocyte-derived macrophages in Alzheimer’s disease models: Effects of aging and amyloid pathology. Aging Cell.

[24] Zattoni M., Mura M.L., Deprez F., Schwendener R.A., Engelhardt B., Frei K., Fritschy J.M. (2011). Brain infiltration of leukocytes contributes to the pathophysiology of temporal lobe epilepsy. J. Neurosci..

[25] Saiwai H., Kumamaru H., Ohkawa Y., Kubota K., Kobayakawa K., Yamada H., Yokomizo T., Iwamoto Y., Okada S. (2013). Ly6C+ Ly6G- Myeloid-derived suppressor cells play a critical role in the resolution of acute inflammation and the subsequent tissue repair process after spinal cord injury. J. Neurochem..

[26] Rosell A., Cuadrado E., Ortega-Aznar A., Hernandez-Guillamon M., Lo E.H., Montaner J. (2008). MMP-9-positive neutrophil infiltration is associated to blood-brain barrier breakdown and basal lamina type IV collagen degradation during hemorrhagic transformation after human ischemic stroke. Stroke.

[27] Justicia C., Panés J., Solé S., Cervera A., Deulofeu R., Chamorro A., Planas A.M. (2003). Neutrophil Infiltration Increases Matrix Metalloproteinase-9 in the Ischemic Brain after Occlusion/Reperfusion of the Middle Cerebral Artery in Rats. J. Cereb. Blood Flow Metab..

[28] Hayashi T., Kaneko Y., Yu S., Bae E., Stahl C.E., Kawase T., van Loveren H., Sanberg P.R., Borlongan C.V. (2009). Quantitative analyses of matrix metalloproteinase activity after traumatic brain injury in adult rats. Brain Res..

[29] Simmons S.B., Liggitt D., Goverman J.M. (2014). Cytokine-Regulated Neutrophil Recruitment Is Required for Brain but Not Spinal Cord Inflammation during Experimental Autoimmune Encephalomyelitis. J. Immunol..

[30] Christy A.L., Walker M.E., Hessner M.J., Brown M.A. (2013). Mast cell activation and neutrophil recruitment promotes early and robust inflammation in the meninges in EAE. J. Autoimmun..

[31] Moxon-Emre I., Schlichter L.C. (2011). Neutrophil Depletion Reduces Blood-Brain Barrier Breakdown, Axon Injury, and Inflammation After Intracerebral Hemorrhage. J. Neuropathol. Exp. Neurol..

[32] Gidday J.M., Gasche Y.G., Copin J.-C., Shah A.R., Perez R.S., Shapiro S.D., Chan P.H., Park T.S. (2005). Leukocyte-derived matrix metalloproteinase-9 mediates blood-brain barrier breakdown and is proinflammatory after transient focal cerebral ischemia. Am. J. Physiol. Circ. Physiol..

[33] Binder D.K., Steinhäuser C. (2021). Astrocytes and Epilepsy. Neurochem. Res..

[34] Clasadonte J., Dong J., Hines D.J., Haydon P.G. (2013). Astrocyte control of synaptic NMDA receptors contributes to the progressive development of temporal lobe epilepsy. Proc. Natl. Acad. Sci. USA.

[35] Small C., Dagra A., Martinez M., Williams E., Lucke-Wold B. (2022). Examining the role of astrogliosis and JNK signaling in post-traumatic epilepsy. Egypt. J. Neurosurg..

[36] Tai T.Y., Warner L.N., Jones T.D., Jung S., Concepcion F.A., Skyrud D.W., Fender J., Liu Y., Williams A.D., Neumaier J.F. (2017). Antiepileptic action of c-Jun N-terminal kinase (JNK) inhibition in an animal model of temporal lobe epilepsy. Neuroscience.

[37] Lu R., Cui S.-S., Wang X.-X., Chen L., Liu F., Gao J., Wang W. (2021). Astrocytic c-Jun N-terminal kinase-histone deacetylase-2 cascade contributes to glutamate transporter-1 decrease and mechanical allodynia following peripheral nerve injury in rats. Brain Res. Bull..

[38] Lucke-Wold B.P., Nguyen L., Turner R.C., Logsdon A.F., Chen Y.W., Smith K.E., Huber J.D., Matsumoto R., Rosen C.L., Tucker E.S. (2015). Traumatic brain injury and epilepsy: Underlying mechanisms leading to seizure. Seizure.

[39] Loscher W., Brandt C. (2010). Prevention or modification of epileptogenesis after brain insults: Experimental approaches and translational research. Pharmacol. Rev..

[40] Belcastro V., Costa C., Galletti F., Autuori A., Pierguidi L., Pisani F., Calabresi P., Parnetti L. (2008). Levetiracetam in newly diagnosed late-onset post-stroke seizures: A prospective observational study. Epilepsy Res..

[41] Klein P., Herr D., Pearl P.L., Natale J., Levine Z., Nogay C., Sandoval F., Trzcinski S., Atabaki S.M., Tsuchida T. (2012). Results of Phase 2 Safety and Feasibility Study of Treatment With Levetiracetam for Prevention of Posttraumatic Epilepsy. Arch. Neurol..

[42] Pearl P.L., McCarter R., McGavin C.L., Yu Y., Sandoval F., Trzcinski S., Atabaki S.M., Tsuchida T., Anker J.V.D., He J. (2013). Results of phase II levetiracetam trial following acute head injury in children at risk for posttraumatic epilepsy. Epilepsia.

[43] Zeng T.-F., Li Y.-H., An D.-M., Chen L., Lei D., Zhang B., Li J.-M., Zhou N. (2014). Effectiveness of levetiracetam use following resective surgery in patients with refractory epilepsy: A prospective observational study. Epilepsy Res..

[44] Kundu B., Lucke-Wold B., Foster C., Englot D.J., Urhie O., Nwafor D., Rolston J.D. (2019). Fornicotomy for the Treatment of Epilepsy: An Examination of Historical Literature in the Setting of Modern Operative Techniques. Neurosurgery.

[45] Racine R.J. (1972). Modification of seizure activity by electrical stimulation: II. Motor seizure. Electroencephalogr. Clin. Neurophysiol..

