# Supplementary material for: Levetiracetam Suppresses the Infiltration of Neutrophils and Monocytes and Downregulates Many Inflammatory Cytokines during Epileptogenesis in Pilocarpine-Induced Status Epilepticus Mice
Source: Int J Mol Sci. 2022 Jul 12;23(14):7671. doi: 10.3390/ijms23147671 (PMC9319101; doi:10.3390/ijms23147671)
Supplement: Supplementary file 1 [file ijms-23-07671-s001.zip › ijms-1789515-supplementary.pdf]

Mouse XL Cytokine Array Coordinates

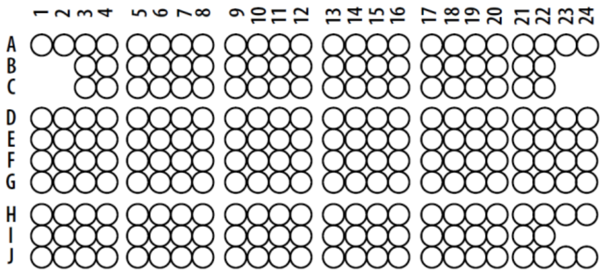

position	Analyte/Control	position	Analyte/Control	position	Analyte/Control	position	Analyte/Control
A1	Reference Spot	C17	CX3CL1/Fractalkine	F5	IGFBP-6	H15	MMP-9
A2		C18		F6		H16	
A3	Adiponectin/Acrp30	C19	CXCL1/KC	F7	IL-1α/IL-1F1	H17	Myeloperoxidase
A4		C20		F8		H18	
A5	Amphiregulin	C21	CXCL2/MIP-2	F9	IL-1β/IL-1F2	H19	Osteopontin (OPN)
A6		C22		F10		H20	
A7	Angiopoietin-1	D1	CXCL9/MIG	F11	IL-1ra/IL-1F3	H21	Osteoprotegerin/TNFRSF 11B
A8		D2		F12		H22	
A9	Angiopoietin-2	D3	CXCL10/IP-10	F13	IL-2	H23	PD-ECGF/Thymidine phosphorylase
A10		D4		F14		H24	
A11	Angiopoietin-like 3	D5	CXCL11/I-TAC	F15	IL-3	I1	PDGF-BB
A12		D6		F16		I2	
A13	BAFF/BLyS/TNFSF13B	D7	CXCL13/BLC/BCA-1	F17	IL-4	I3	Pentraxin 2/SAP
A14		D8		F18		I4	
A15	C1q R1/CD93	D9	CXCL16	F19	IL-5	I5	Pentraxin 3/TSG-14
A16		D10		F20		I6	
A17	CCL2/JE/MCP-1	D11	Cystatin C	F21	IL-6	I7	Periostin/OSF-2
A18		D12		F22		I8	
A19	CCL3/CCL4/MIP-1a/b	D13	DKK-1	F23	IL-7	I9	Pref-1/DLK-1/FA1
A20		D14		F24		I10	
A21	CCL5/RANTES	D15	DPPIV/CD26	G1	IL-10	I11	Proliferin
A22		D16		G2		I12	
A23	Reference Spot	D17	EGF	G3	IL-11	I13	Proprotein Convertase 9/PCSK9
A24		D18		G4		I14	
B3	CCL6/C10	D19	Endoglin/CD105	G5	IL-12 p40	I15	RAGE
B4		D20		G6		I16	
B5	CCL11/Eotaxin	D21	Endostatin	G7	IL-13	I17	RBP4
B6		D22		G8		I18	
B7	CCL12/MCP-5	D23	Fetuin A/AHSG	G9	IL-15	I19	Reg3G
B8		D24		G10		I20	
B9	CCL17/TARC	E1	FGF acidic	G11	IL-17A	I21	Resistin
B10		E2		G12		I22	
B11	CCL19/MIP-3β	E3	FGF-21	G13	IL-22	J1	Reference Spot
B12		E4		G14		J2	
B13	CCL20/MIP-3α	E5	Flt-3 Ligand	G15	IL-23	J3	E-Selectin/CD62E
B14		E6		G16		J4	
B15	CCL21/6Ckine	E7	Gas 6	G17	IL-27 p28	J5	P-Selectin/CD62P
B16		E8		G18		J6	
B17	CCL22/MDC	E9	G-CSF	G19	IL-28A/B	J7	Serpin E1/PAI-1
B18		E10		G20		J8	
B19	CD14	E11	GDF-15	G21	IL-33	J9	Serpin F1/PEDF
B20		E12		G22		J10	
B21	CD40/TNFRSF5	E13	GM-CSF	G23	LDL R	J11	Thrombopoietin
B22		E14		G24		J12	
C3	CD160	E15	HGF	H1	Leptin	J13	TIM-1/KIM-1/HAVCR
C4		E16		H2		J14	
C5	Chemerin	E17	ICAM-1/CD54	H3	LIF	J15	TNF-α
C6		E18		H4		J16	
C7	Chitinase 3-like 1	E19	INF-γ	H5	Lipocalin-2/NGAL	J17	VCAM-1/CD106
C8		E20		H6		J18	
C9	Coagulation Factor III/Tissue Factor	E21	IGFBP-1	H7	LIX	J19	VEGF
C10		E22		H8		J20	
C11	Complement Component C5/C5a	E23	IGFBP-2	H9	M-CSF	J21	WISP-1/CCN4
C12		E24		H10		J22	
C13	Complement Factor D	F1	IGFBP-3	H11	MMP-2	J23	Negative Control (no antibody)
C14		F2		H12		J24	
C15	C-Reactive Protein/CRP	F3	IGFBP-5	H13	MMP-3		
C16		F4		H14			

Figure S1. Schematic representation of the Mouse XL Cytokine Array.
Capture and control antibodies have been spotted in duplicate on nitrocellulose membranes. The table shows a list and coordinates of analytes and controls.

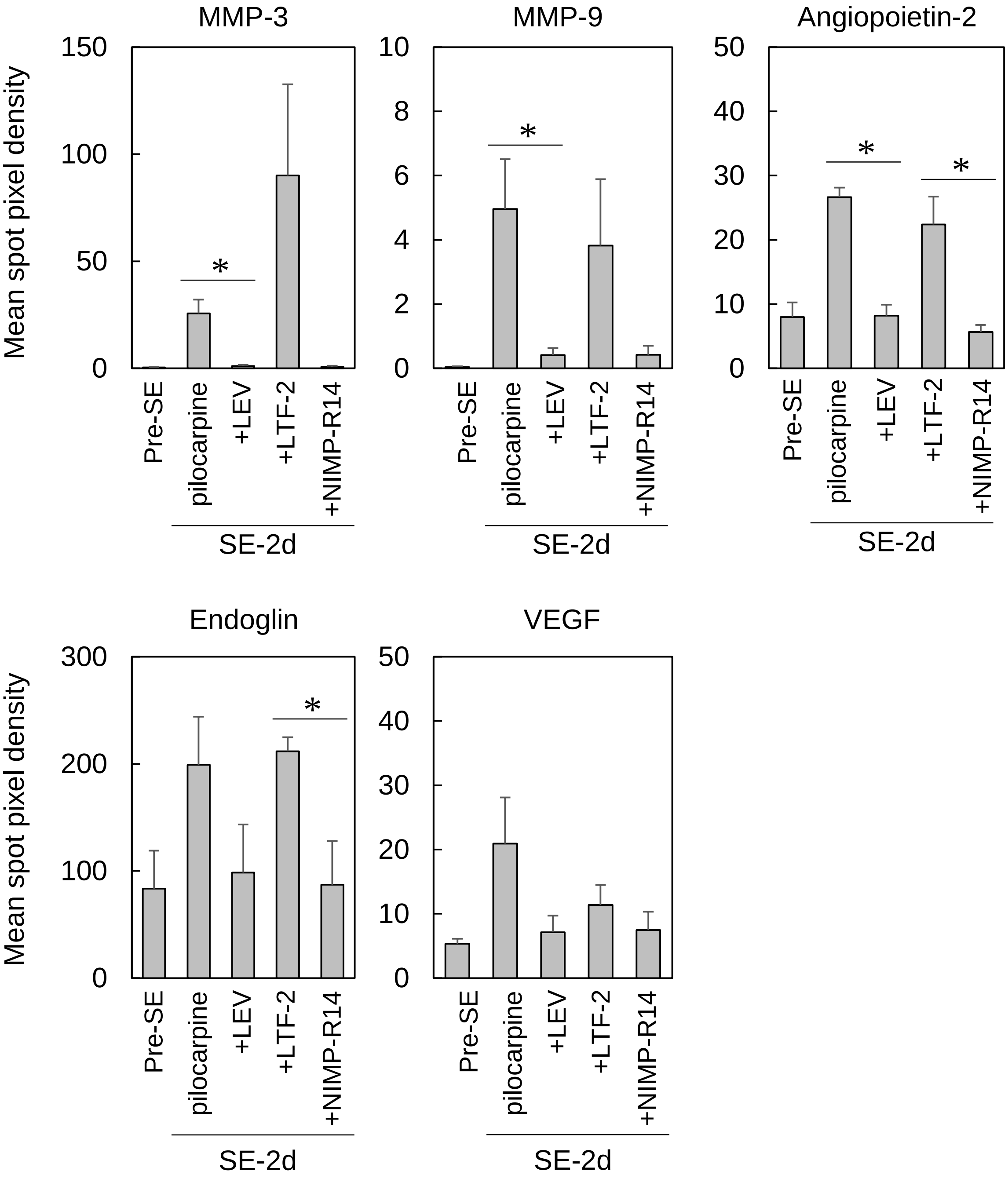

Figure S2. Expression of cytokines associated with BBB disruption.
The expression levels of cytokines were determined by measuring pixel intensities of spots in cytokine arrays. *n* = 3 mice per group. Data are means ± SEMs. *$p < 0.05$, unpaired *t* test.

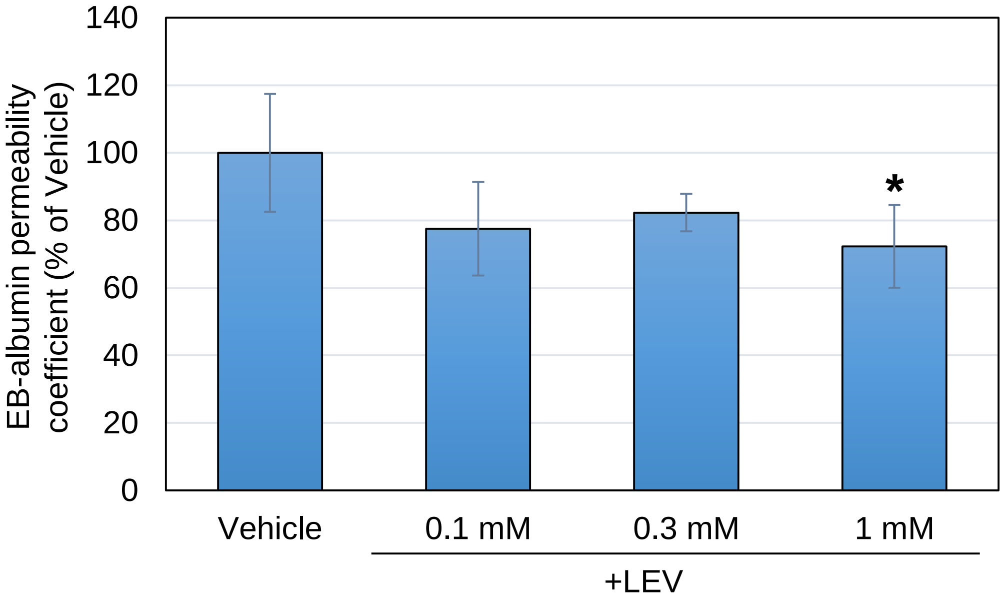

Figure S3 The influence of LEV treatment on the permeability of EBA in *in vitro* BBB model. The permeability of EBA in the adult rat brain capillary endothelial cells BBB model. Four independent experiments were performed. Data were analyzed for significant differences by one way ANOVA post-hoc Dunnett's test. * P=0.016 significantly different from each corresponding control.

Supplementary Materials and Methods.

In vitro evaluation of barrier functions

Rat brain capillary endothelial cells obtained from 8 week-old Wister rats were seeded on the insides of inserts (Corning, Midland, MI, USA) and maintained in culture medium previously described (PMID: 23523792, DOI: 10.1016/j.bbrc.2013.03.036). The brain capillary endothelial cells were treated with 0.1, 0.3, and 1 mM of LEV for 24 h into the inside of the insert. After that, the permeability coefficients to Evans blue albumin (EBA) were measured for evaluating barrier function in the endothelial cells layers as previously described (PMID: 23523792, DOI: 10.1016/j.bbrc.2013.03.036).
